# Analysis of antimicrobial effects of a contactless, indirect cold atmospheric plasma-aerosol method for germ reduction on surfaces: an *in vitro* and *in vivo* study

**DOI:** 10.3389/fmicb.2025.1668987

**Published:** 2025-09-18

**Authors:** Tom Schaal, Ulrich Schmelz, Gilbert Hämmerle, Robert Fuchs, Timon Schorling, Sandra Kurras, Marc Koene, Tim Tischendorf

**Affiliations:** ^1^Faculty of Health and Healthcare Sciences, University of Applied Sciences Zwickau, Zwickau, Germany; ^2^Department of Nursing and Health, University of Fulda, Fulda, Germany; ^3^Wound Outpatient Clinic LKH Bregenz, Bregenz, Austria; ^4^Research and Development, WK-MedTec GmbH, Bückeburg, Germany; ^5^Lüsche Veterinary Clinic, Bakum, Germany

**Keywords:** cold plasma, wound treatment, chronic wounds, germ reduction, CAP, CAP-A

## Abstract

Cold Atmospheric Plasma-Aerosol (CAP-A) technology has emerged as a novel, contactless approach for antimicrobial treatment. This study evaluates the *in vivo* efficacy of CAP-A in reducing microbial contamination on human skin, building on obligatory *in vitro* testing. *In vitro* results demonstrated consistent 3–4.5 log unit microbial reductions across five standard organisms. *In vivo* evaluation using *Escherichia coli* revealed a mean log reduction factor of 4.77 (SD ± 0.44), exceeding the 4-log threshold considered clinically relevant. Notably, CAP-A showed comparable efficacy to an alcohol-based reference disinfection method (*p* = 0.134), without associated drawbacks such as thermal effects or ozone accumulation. Results suggest that CAP-A offers equivalent *in vivo* efficacy compared to previously documented CAP methods while minimizing tissue damage, thermal changes, and discomfort. The results underscore the potential of CAP-A as an effective and tolerable alternative to established CAP approaches, warranting further comparative research under standardized conditions. Future studies should examine both CAP and CAP-A technologies, broadening the spectrum of tested microorganisms, incorporating additional parameters, and rigorously assessing benefits and risks. This research could elucidate the underlying mechanisms driving differences in efficacy and side effect profiles, ultimately contributing to the optimization of plasma-based treatments in clinical and industrial settings.

## 1 Introduction

Plasma, the fourth state of matter, is a partially ionized gas composed of charged particles. In medical applications, Cold Atmospheric Plasmas (CAP) are increasingly explored for their antimicrobial effects. Particularly, plasmas generated from oxygen and water vapor yield reactive oxygen species (ROS) capable of modifying surfaces, liquids, and even ambient air through energy transfer during ionization processes (Lerouge et al., 2001; Graves and Bauer, 2018; Ehlbeck et al., 2011). Until recently, most research has focused on direct plasma applications, where plasma is applied directly to the target surface. These methods have shown strong microbiocidal effects in wound sanitation, effectively reducing bacterial load regardless of wound size, location or cofactors (e.g., tobacco abuse, cardiovascular diseases) (Braný et al., 2020; Apelqvist et al., 2024; Daeschlein et al., 2015). Notably, Daeschlein et al. (2015) demonstrated *in vivo* efficacy against both Gram-positive and Gram-negative bacteria, including resistant strains such as MRSA and MRGN, indicating that cold plasma damages microbial structures in a way that bypasses typical resistance mechanisms.

A drop in wound pH after plasma treatment further supports its antimicrobial efficacy, as pathogenic bacteria typically alkalize the wound environment. CAP treatment restores the acidic milieu necessary for optimal healing (Hämmerle et al., 2023). Moreover, studies report significantly accelerated wound healing and increased healing rates, up to 60% higher compared to supportive therapy alone (Abu Rached et al., 2023; Strohal et al., 2022; Stratmann et al., 2020; Isbary et al., 2012; Isbary et al., 2010; Schachl et al., 2025). According to Strohal et al. (2022), these improvements are independent of wound type or location. The observed efficacy is attributed primarily to the antimicrobial action, suggesting that indirect plasma methods may yield similar results.

Building on these findings, a novel indirect plasma approach - Cold Atmospheric Plasma-Aerosol (CAP-A) - has been introduced. CAP-A combines reactive species from CAP with nebulized water, enabling antimicrobial action without direct contact between the plasma source and the treated surface (Tischendorf et al., 2024; Schaal and Schmelz, 2024). Energy input is limited to plasma generation, making the method safer and more flexible in clinical applications. Indirect methods like CAP-A target the transient skin flora via redox disruption and induce a “selective short circuit” in microbial membranes, collapsing the membrane potential and disabling transmembrane transport (Braný et al., 2020).

Importantly, these effects are highly selective. Human cells remain unaffected due to protective antioxidant enzymes such as catalase, superoxide dismutase, and alpha-1-antitrypsin (Masur et al., 2018). Toxicological evaluations of CAP-A, including the Ames test (EN ISO 10993-3) and cytotoxicity tests (EN ISO 10993-5), confirm its safety profile. No mutagenic or cytotoxic effects were detected in bacteria or human cells, respectively. Clinical use of direct CAP devices has also shown no adverse effects, reinforcing the biological selectivity and clinical safety of this emerging technology (Masur et al., 2018).

The analysis builds on previous work demonstrating the microbicidal efficacy of CAP-A technology in diverse experimental and practical settings including the disinfection of thermolabile medical devices under routine hospital conditions (Schaal et al., 2025) and the successful treatment of clinical infections in veterinary patients (Kurras et al., 2025). This study represents a first EN 1500-compliant *in vivo* investigation of CAP-A on human skin using a specified test germ for clear statistical and clinical evidence. Further, the test setting combines two standards for assessing plasma techniques. EN 1500 presents the closest standard to CAP-A for surface-active reduction of microbial load in a clinical setting and DIN SPEC 91315 defines the measurement of inactivation potency of plasma sources. The two norms applied for both chemical disinfectants (EN 1500) and plasma devices (DIN SPEC 91315) demonstrate the reliability of microbial inactivation results of CAP-A. This article critically examines how effective is the CAP-A procedure in reducing microbial contamination on human skin under *in vivo* conditions, following the obligatory preliminary *in vitro* testing as required by best practice. Furthermore, directly comparing its antimicrobial performance with the established alcohol-based reference method, this work addresses a critical gap between laboratory-based findings and clinically relevant, standardized testing.

How does the antimicrobial performance of CAP-A, evaluated in alignment with the reference alcohol-based disinfection method under EN 1500 conditions, compare with the documented efficacy of existing CAP procedures in current literature? Can these findings provide insights into the future role and integration of CAP and CAP-A methods in clinical treatment practices?

## 2 Subsections relevant for the subject

### 2.1 Working principle

The PLASMO®HEAL device (WK-MedTec GmbH, Bückeburg) operates via an indirect plasma method. A defined alternating voltage (1.45 kV, 38 kHz, sinusoidal) generates cold plasma reaction products, primarily hydroxyl radicals, from ambient air, while only negligible amounts of ozone are produced. Distilled water (stabilized according to German pharmacopoeia for hygienic reasons) is nebulized ultrasonically and activated by the plasma-enriched air, thereby increasing the electrophysical potential of the aerosol without altering its material composition. The resulting aerosol (CAP-A) is then applied to the targeted surface (e.g., skin or wounds) at a distance of 7.5 cm for three minutes, as specified in the manufacturer’s operating instructions to ensure homogeneous distribution across the treatment area and optimal antimicrobial efficacy. In accordance with EN 1500 test conditions, post-treatment sampling in this study was performed 40 min after application, allowing a preliminary assessment of the medium-term persistence of the antimicrobial effect.

### 2.2 *In vitro* testing

Microbial reduction efficacy was assessed *in vitro* against five standard organisms (*Staphylococcus aureus* ATCC 6538, *Staphylococcus epidermidis* ATCC 14990, *Escherichia coli* NCTC 10538, *Pseudomonas aeruginosa* ATCC 10145, and *Candida albicans* ATCC 10321) according to DIN 91315:2014, with each organism tested three times. Identification of the organisms was confirmed by Gram staining, metabolic profiling, and indole production. Cultures were grown on Caso agar in 60-mm Petri dishes and adjusted to McFarland standard 8 with sterile, pyrogen-free 0.9% NaCl. A microbial suspension was applied to stainless steel specimens (100 × 10 × 1 mm; surface roughness 100 μm) using a sterile swab, followed by a five-second immersion, drainage, and two-hour air drying. The specimens were then exposed to the PLASMO®HEAL aerosol for three minutes. Microorganisms were recovered by vortexing each sample in 10 mL sterile 0.9% NaCl for 30 s; serial tenfold dilutions (effective dilution from 100 to 10^−3^) were plated (0.1 mL per dilution) alongside untreated controls. Following incubation, colony counts (from plates with 10–100 colonies) were used to calculate microbial loads and determine the log reduction factors.

### 2.3 *In vivo* testing

*In vivo* antimicrobial efficacy was evaluated in accordance with EN 1500 using *Escherichia coli* NCTC 10538, a non-pathogenic reference strain specified for standardized hygienic handrub testing. Eighteen subjects participated as part of routine hospital hand hygiene quality assurance (not as a clinical trial). Both hands were contaminated by immersing the fingers in a test suspension for five seconds, then drained. The left hand provided a pre-treatment bacterial count, while the right hand, after contamination, was exposed to the PLASMO®HEAL aerosol for three minutes before post-treatment sampling. Serial dilutions and culture methods analogous to the *in vitro* procedure produced log reduction factors. *E. coli*, an apathogenic yet resilient organism, served as a suitable surrogate for pathogenic microbes. The number of test subjects (n = 18) was determined in accordance with the requirements of EN 1500 in order to ensure sufficient statistical significance for the evaluation of hygienic hand disinfection procedures. All procedures were conducted in accordance with relevant guidelines; participants provided written informed consent, and an ethical waiver was secured from the institutional review board (recruitment: January 2–9, 2023).

### 2.4 Statistical analysis

Descriptive and inferential statistics were performed using IBM Statistics Version 30 for Mac. Given the small sample size, the Shapiro–Wilk test assessed normality for the log-CFU parameter (pre: *p* = 0.025; post: *p* = 0.191) and for the log-CFU reduction factor (*p* < 0.001), with normal distribution confirmed only for post-treatment log-CFU values. Simulation studies support the *t*-test’s robustness to normality violations (Rasch and Guiard, 2004). Accordingly, a paired samples *t*-test (*α* = 5%, two-tailed) compared pre- and post-treatment log-CFU values in the *in vivo* testing, while an independent samples *t*-test (*α* = 5%, two-tailed), preceded by Levene’s test for equality of variances (*p* = 0.0502), compared the log-CFU reduction factors between the CAP-A and the alcohol-based reference disinfection. The complete data set is included as an e-supplement.

## 3 Results

Detailed analyses of the *in vitro* results (Table 1) and in vivo outcomes (Table 2) provide a comprehensive assessment of the dynamic effects of this innovative wound treatment method. In the *in vivo* evaluation employing the CAP-A procedure, the mean log CFU prior to application was 7.23 (SD ± 0.59), which decreased to 2.52 (SD ± 0.38) post-procedure, yielding a mean log reduction factor of 4.77 (SD ± 0.44). A paired samples *t*-test confirmed that the reduction in log CFU values was statistically significant (*p* < 0.001). Furthermore, comparison of the log CFU reduction factors between the CAP-A treatment and the alcohol-based reference disinfection revealed no significant difference, as indicated by Levene’s test for homogeneity of variances (*p* = 0.0502) and an independent samples *t*-test (*p* = 0.134). Based on the *in vivo* results, the CAP-A procedure achieved a disinfection efficacy exceeding a 4-log reduction, thereby confirming its effectiveness. Moreover, the statistical analyses indicated no significant difference between the CAP-A method and the alcohol-based reference disinfection, suggesting that the two are equivalent in performance, even though current EN 1500 standards are not yet tailored to accommodate such innovative approaches.

**Table 1:** Results of the *in vitro* test of the germ-reducing effect of the CAP-A method.

No.	V1	V2	CfU/Agar plate	CfU/Approach	Dilution	CfU/Proband	Log CfU/Proband	Log reductions factor
*Staphylococcus aureus* 3 min								
1.1. A-3	10.0 mL	0.1 mL	87	8,700	10,000	87,000,000	**7.94**	
1.1. B-3	10.0 mL	0.1 mL	28	2,800	1	2,800	3.45	4.49
1.2. A-3	10.0 mL	0.1 mL	56	5,600	10,000	56,000,000	7.75	
1.2. B-3	10.0 mL	0.1 mL	26	2,600	1	2,600	3.41	4.33
1.3. A-3	10.0 mL	0.1 mL	78	7,800	10,000	78,000,000	7.89	
1.3. B-3	10.0 mL	0.1 mL	22	2,200	1	2,200	3.34	4.55
*Staphylococcus epidermidis* 3 min								
1.1. A-3	10.0 mL	0.1 mL	56	5,600	10,000	56,000,000	7.75	
1.1. B-3	10.0 mL	0.1 mL	31	3,100	1	3,100	3.49	4.26
1.2. A-3	10.0 mL	0.1 mL	44	4,400	10,000	44,000,000	7.64	
1.2. B-3	10.0 mL	0.1 mL	25	2,500	1	2,500	3.40	4.25
1.3. A-3	10.0 mL	0.1 mL	76	7,600	10,000	76,000,000	7.88	
1.3. B-3	10.0 mL	0.1 mL	37	3,700	1	3,700	3.57	4.31
*Escherichia coli* 3 min								
1.1. A-3	10.0 mL	0.1 mL	92	9,200	10,000	92,000,000	7.96	
1.1. B-3	10.0 mL	0.1 mL	54	5,400	1	5,400	3.73	4.23
1.2. A-3	10.0 mL	0.1 mL	78	7,800	10,000	78,000,000	7.89	
1.2. B-3	10.0 mL	0.1 mL	34	3,400	1	3,400	3.53	4.36
1.3. A-3	10.0 mL	0.1 mL	65	6,500	10,000	65,000,000	7.81	
1.3. B-3	10.0 mL	0.1 mL	39	3,900	1	3,900	3.59	4.22
*Pseudomonas aeruginosa* 3 min								
1.1. A-3	10.0 mL	0.1 mL	72	7,200	10,000	72,000,000	7.86	
1.1. B-3	10.0 mL	0.1 mL	26	2,600	1	2,600	3.41	4.44
1.2. A-3	10.0 mL	0.1 mL	61	6,100	10,000	61,000,000	7.79	
1.2. B-3	10.0 mL	0.1 mL	34	3,400	1	3,400	3.53	4.25
1.3. A-3	10.0 mL	0.1 mL	93	9,300	10,000	93,000,000	7.97	
1.3. B-3	10.0 mL	0.1 mL	45	4,500	1	4,500	3.65	4.32
*Candida albicans* 3 min								
1.1. A-3	10.0 mL	0,1 mL	78	7,800	10,000	7,800,000	6.89	
1.1. B-3	10.0 mL	0,1 mL	31	3,100	1	3,100	3.49	3.40
1.2. A-3	10.0 mL	0,1 mL	59	5,900	10,000	59,000,000	7.77	
1.2. B-3	10.0 mL	0,1 mL	27	2,700	1	2,700	3.43	4.34
1.3. A-3	10.0 mL	0,1 mL	86	8,600	10,000	86,000,000	7.93	
1.3. B-3	10.0 mL	0,1 mL	29	2,900	1	2,900	3.46	4.47

No., identification of the test run, where the first two digits stand for the test person number; ‘A’, test run before disinfection (initial bacterial count); ‘B’, test run after disinfection (initial bacterial count); V1, total volume approach to leaching; V2, volume of the batch from V1, which was applied to the agar plate; CfU, colony forming unit.

**Table 2:** Results of the in vivo test of the germ-reducing effect of the CAP-A method.

No.	V1	V2	CfU/Agar plate	CfU/Approach	Dilution	CfU/Proband	Log CfU/Proband	Log reductions factor	Mean values Log RF
01 before	10.0 mL	0.1 mL	46	4,600	10,000	46,000,000	7.66		
01 after	10.0 mL	0.1 mL	3	300	1	300	2.48	5.19	5.19
02 before	10.0 mL	0.1 mL	18	1,800	10,000	18,000,000	7.26		
02 after	10.0 mL	0.1 mL	5	500	1	500	2.70	4.56	4.56
03 before	10.0 mL	0.1 mL	16	1,600	100,000	160,000,000	8.20		
03 after	10.0 mL	0.1 mL	14	1,400	1	1,400	3.15	5.06	5.06
04 before	10.0 mL	0.1 mL	23	2,300	1,000	2,300,000	6.36		
04 after	10.0 mL	0.1 mL	2	200	1	200	2.30	4.06	4.06
05 before	10.0 mL	0.1 mL	84	8,400	10,000	84,000,000	7.92		
05 after	10.0 mL	0.1 mL	5	500	1	500	2.70	5.23	5.23
06 before	10.0 mL	0.1 mL	15	1,500	10,000	15,000,000	7.18		
06 after	10.0 mL	0.1 mL	3	300	1	300	2.48	4.70	4.70
07 before	10.0 mL	0.1 mL	4	400	10,000	400,000	5.60		
07 after	10.0 mL	0.1 mL	1	100	1	100	2.00	3.60	3.60
08 before	10.0 mL	0.1 mL	92	9,200	10,000	92,000,000	7.96		
08 after	10.0 mL	0.1 mL	17	1,700	1	1700	3.23	4.73	4.73
09 before	10.0 mL	0.1 mL	39	3,900	10,000	39,000,000	7.59		
09 after	10.0 mL	0.1 mL	5	500	1	500	2.70	4.89	4.89
10 before	10.0 mL	0.1 mL	24	2,400	10,000	24,000,000	7.38		
10 after	10.0 mL	0.1 mL	1	100	1	100	2.00	5.38	5.38
11 before	10.0 mL	0.1 mL	17	1,700	10,000	17,000,000	7.23		
11 after	10.0 mL	0.1 mL	5	500	1	500	2.70	4.53	4.53
12 before	10.0 mL	0.1 mL	11	1,100	10,000	11,000,000	7.04		
12 after	10.0 mL	0.1 mL	1	100	1	100	2.00	5.04	5.04
13 before	10.0 mL	0.1 mL	26	2,600	10,000	26,000,000	7.41		
13 after	10.0 mL	0.1 mL	2	200	1	200	2.30	5.11	5.11
14 before	10.0 mL	0.1 mL	14	1,400	10,000	14,000,000	7.15		
14 after	10.0 mL	0.1 mL	4	400	1	400	2.60	4.54	4.54
15 before	10.0 mL	0.1 mL	14	1,400	10,000	14,000,000	7.15		
15 after	10.0 mL	0.1 mL	1	100	1	100	2.00	5.15	5.15
16 before	10.0 mL	0.1 mL	39	3,900	10,000	39,000,000	7.59		
16 after	10.0 mL	0.1 mL	9	900	1	900	2.95	4.64	4.64
17 before	10.0 mL	0.1 mL	21	2,100	10,000	21,000,000	7.32		
17 after	10.0 mL	0.1 mL	3	300	1	300	2.48	4.85	4.85
18 before	10.0 mL	0.1 mL	14	1,400	10,000	14,000,000	7.15		
18 after	10.0 mL	0.1 mL	4	400	1	400	2.60	4.54	4.54
							Mean value log RF	4.77	
							Median log RF	4.79	

No., identification of the test run; V1, total volume approach to leaching; V2, volume of the batch from V1, which was applied to the agar plate; CfU, colony forming unit; RF, reduction factor.

## 4 Discussion

This study evaluated the *in vivo* antimicrobial efficacy of the CAP-A procedure using the WK-MedTec GmbH PLASMO®HEAL device against the test organism *E. coli*. Our findings demonstrated a mean log reduction factor of 4.77, indicating a robust antimicrobial effect that is statistically significant. When considered alongside previous reports on CAP-based interventions, these results suggest that CAP-A can achieve antimicrobial reductions in a range comparable to those documented for other CAP methods, despite differences in organisms tested, exposure times, device configurations, and application sites across studies. For instance, Zimmermann (Zimmermann et al., 2011) achieved a mean log reduction factor of over 3 CFU *in vitro*, while Boekema et al. reported an in vivo log reduction factor of 2.9 (Boekema et al., 2021). Similarly, Perni et al. (2007) demonstrated a reduction factor of 2.5 following a 2.5-min CAP treatment. In contrast, our study’s achieved value of 4.77 supports the consideration of CAP-A as a viable, EN 1500-compliant alternative to direct CAP. Lan et al. (2024) have shown that both CAP and CAP-A techniques are capable of producing high log reduction factors. However, where CAP procedures have been associated with heightened power input and consequential effects, such as increased production of nitrate and ozone and augmented local temperature (Daeschlein et al., 2012; Gelker et al., 2019), CAP-A appears to achieve comparable bacterial inactivation while potentially reducing these secondary side effects. In addition to antimicrobial efficacy, potential skin-related adverse effects were evaluated. Skin irritation testing performed in accordance with DIN EN ISO 10993-23:2021–10 demonstrated no evidence of erythema or edema at any observation point, resulting in a Primary Irritation Index (P. I. I.) of 0.0. These findings indicate that CAP-A treatment, when applied as described in this study, is unlikely to induce skin irritation, supporting its potential suitability for repeated or prolonged clinical use. In particular, CAP-A’s lower energy requirement, combined with its high antimicrobial efficacy, positions it as a promising alternative in applications where minimizing thermal alterations and chemical by-products is desirable.

Although the current corpus of literature does not provide an exhaustive depiction of the pooled log reduction factors achievable by CAP procedures, the results presented here underscore that modern CAP-A technologies are not necessarily inferior. On the contrary, the promising log reduction factor of 4.77 achieved in our study supports the hypothesis that CAP-A may serve as an efficacious alternative to traditional CAP methods without the concomitant drawbacks of increased reactive species production and thermal side effects.

In accordance with EN 1500 and DIN SPEC 91315, *E. coli* NCTC 10538 was selected for the *in vivo* evaluation to ensure standardization and comparability with established disinfectant testing protocols. The *in vitro* component was designed to extend this evaluation to a broader microbial spectrum, including Gram-positive and Gram-negative bacteria, thereby enabling a representative evaluation within the framework of recognized validation standards for plasma-based and chemical disinfectants. In summary, our findings indicate that CAP-A, by achieving a significant in vivo log reduction factor while mitigating the adverse effects noted in conventional CAP treatment, holds considerable potential as an alternative antimicrobial strategy. This study provides a meaningful impetus for further investigation into the comparative efficiencies and mechanistic underpinnings of CAP versus CAP-A procedures.

## 5 Limitations and future research

This study has certain limitations that should be considered when interpreting the results. The in vivo evaluation was performed exclusively on healthy volunteers under standardized EN 1500 conditions, which may not fully reflect the complexity of clinical environments such as chronic wound care or high-contamination scenarios. The use of a single non-pathogenic test organism (*Escherichia coli*) provides a controlled model but does not account for the spectrum of microorganisms encountered in practice, including resistant strains, spore-formers, or fungal biofilms. In addition, the relatively small sample size may limit the detection of inter-individual variability, and the study did not investigate possible cumulative effects or durability of microbial reduction over time. Future research should therefore focus on assessing CAP-A performance in patient populations with different skin conditions, compromised immunity, or existing infections. Moreover, studies under realistic clinical workflow constraints, including integration within existing hygiene protocols, will be important. In this study, post-treatment microbial counts were obtained 40 min after the 3-min CAP-A application, as recommended in the device’s operating protocol. While this interval under EN 1500 conditions offers a preliminary indication of medium-term persistence, it does not substitute for a dedicated long-term evaluation of antimicrobial durability.

Given these observations, we advocate for further comparative research that examines both CAP and CAP-A technologies in parallel. Future studies should broaden the spectrum of tested microorganisms, incorporate additional parameters such as energy consumption and patient comfort, and rigorously assess both the short- and long-term benefits and risks in extended follow up studies associated with these antimicrobial methodologies. Such research could elucidate the underlying mechanisms driving the differences in efficacy and side effect profiles, ultimately contributing to the optimization of plasma-based treatments in both clinical and industrial settings.

## 6 Conclusion

This study demonstrates that the CAP-A method, as applied with the PLASMO®HEAL device, achieves robust antimicrobial activity both *in vitro* and *in vivo*. CAP-A achieved consistently high log reduction factors for five standard microorganisms in vitro, and in vivo tests against *Escherichia coli* showed a mean reduction of 4.77, exceeding the clinically relevant threshold of 4. Importantly, CAP-A’s performance is statistically equivalent to that of an alcohol-based reference method, while in addition avoiding potential drawbacks of conventional CAP techniques. These results position CAP-A as a promising alternative antimicrobial strategy with high efficacy, good safety, and broader clinical applicability. By combining contactless application and inactivation of microorganisms, CAP-A offers advantages in situations where tissue preservation and patient comfort are paramount. Future research should expand the range of target microorganisms, evaluate long-term safety, and compare CAP-A with other new disinfection technologies under standardized clinical conditions. The integration of CAP-A as standardized adjunctive therapeutic step into clinical protocols could represent an effective, patient-friendly innovation. CAP-A has the potential of improving infection control and wound care in human and veterinary medicine.

## Data Availability

The original contributions presented in the study are included in the article/supplementary material, further inquiries can be directed to the corresponding author.
